# Social prescribing within five European countries: a protocol of a cross-country qualitative analysis

**DOI:** 10.1136/bmjopen-2025-112887

**Published:** 2026-01-12

**Authors:** Stephanie Tierney, Debra Westlake, Farhad Rezvani, Daniela Rojatz, Juliane Köberlein-Neu, Trutz Bommhardt, Sónia Dias, Maria João Marques, Donata Kurpas, Hendrik Napierala, Wolfram Herrmann, K Husk

**Affiliations:** 1 Nuffield Department of Primary Care Health Sciences, University of Oxford, Oxford, UK; 2 Institute of General Practice and Family Medicine, Charité - Universitätsmedizin Berlin, Berlin, BE, Germany; 3 Department for Health, Society and Equity, Austrian National Public Health Institute, Vienna, Austria; 4 Center for Health Economics and Health Services Research, University of Wuppertal, Wuppertal, Germany; 5 Center for Health Economics and Health Services Research, University of Wuppertal Schumpeter Faculty of Management and Economics - School of Business and Economics, Wuppertal, Germany; 6 National School of Public Health, Public Health Research Centre, NOVA University Lisbon, Lisbon, Lisbon, Portugal; 7 Department of Nursing, Wrocław Medical University, Wrocław, Poland; 8 Peninsula Medical School, University of Plymouth, Plymouth, England, UK

**Keywords:** Delivery of Health Care, Integrated, General Practice, Health Equity, Psychosocial Intervention, QUALITATIVE RESEARCH

## Abstract

**Abstract:**

**Introduction:**

Social prescribing is an approach to addressing non-medical issues affecting people’s health and well-being (eg, loneliness, housing or financial problems). It has gained international traction over recent years as complementary to medical care. A larger research project, comparing social prescribing across European countries, is considering how to tailor provision for the following groups: (a) LGBTIQ+persons, (b) refugees and first-generation immigrants and (c) older adults living alone. As part of this research, a qualitative study will address the question: *What are the enabling and limiting factors associated with implementing social prescribing, across different European countries, from the perspective of key stakeholders?*

**Methods and analysis:**

Five European countries (Austria, England, Germany, Poland, Portugal) will be involved. Researchers from each country will conduct approximately 20 semi-structured interviews (total number will be 100). Interviewees will be people receiving, delivering, managing and funding/commissioning social prescribing. Interviews will be audio-recorded and transcribed. A cross-country analysis will be undertaken; framework analysis will support this process, with a chart developed in Excel in which data from across the five countries is summarised by the researchers involved. Summaries will be based on a thematic framework that researchers from the five countries develop together after initially analysing their own data.

**Ethics and dissemination:**

Ethical approval was initially secured through the University of Oxford’s Medical Sciences Interdivisional Research Ethics Committee (IDREC 1806086) for data collection in England. This approved application was then used to secure ethics approval in Austria (through Ludwig Boltzmann Gesellschaft), Germany (through Bergische Universität Wuppertal), Poland (through Wroclaw Medical University) and Portugal (through NOVA University of Lisbon). Dissemination will include an academic journal article and presentation at relevant conferences. It will also include short videos, written summaries/policy briefs and an infographic.

This project has received funding from the European Union’s Horizon Europe Research and Innovation Programme under grant agreement No 101155873. Views and opinions expressed are, however, those of the author(s) only and do not necessarily reflect those of the European Union or the European Health and Digital Executive Agency (HADEA). Neither the European Union nor the granting authority can be held responsible for them.

**STRENGTHS AND LIMITATIONS OF THIS STUDY**

- The study will allow for an understanding of social prescribing delivery, tailored for different populations, from a multi-national perspective.
- It provides the opportunity to consider taken-for-granted assumptions about social prescribing delivery by involving researchers and data collection across five European countries.
- Shared topic guides will be used across the countries involved; they will be revised at regular meetings involving researchers from these five countries.
- Challenges may be encountered in recruiting relevant participants in all countries as social prescribing is more established in some than others.
- Cross-cultural variations in understanding of key concepts will need to be considered as part of the analysis.

## Introduction

**Figure 1:**
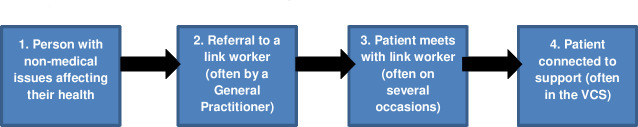
The social prescribing pathway.^6^ VCS, voluntary-community sector.

 Social determinants of health, such as loneliness, housing or financial difficulties, can have a negative impact on people’s quality of life and their mental and physical well-being.^1^ Accessing non-medical support for such problems, often through the voluntary-community sector (VCS), is a key aim of social prescribing. It is defined as ‘a means for trusted individuals in clinical and community settings to identify that a person has non-medical, health-related social needs and to subsequently connect them to support and services within the community by co-producing a social prescription—a non-medical prescription, to improve health and well-being and to strengthen community connections’.^2^ As part of social prescribing, individuals called link workers or social prescribers (other terms may be used^3^) assist people in connecting with relevant non-medical support.^4 5^ These individuals form part of the social prescribing pathway^6^ outlined in figure 1.

Evidence suggests that social prescribing enables people to develop their social networks, to access practical assistance with issues such as finances or housing problems, and can improve mental well-being.^7^,^10^ This has led to its national roll-out in England within primary care, and a growing interest in its delivery across the globe, with several European countries taking up social prescribing to differing extents.^11^,^13^

Social prescribing may need to be tailored to meet the needs of specific populations who could benefit and are not using or accessing it. This is being explored in a Horizon Europe research project (called Social Prescribing-EU [SP-EU]) involving 10 European countries, which is focusing on the following populations:

- LGBTIQ+persons (lesbian, gay, bisexual, transgender, queer or questioning, intersex and asexual, aromantic or agender).
- Refugees and first-generation immigrants.
- Older adults (≥65 years) living alone.

These groups have been selected because they experience (a) barriers to accessing health and social care and/or (b) increased risk of health-related social problems.^14^,^22^ This research (SP-EU) involves:

1. Co-creating social prescribing delivery and implementation, tailored to meet the needs, resources and social context of the three groups listed above.
2. Assessing the effectiveness of tailored social prescribing in terms of improving access to non-clinical support among people from the three groups listed above across European countries.
3. Exploring ways to implement social prescribing that make it accessible.

Within this paper, we describe the proposed approach to be taken to address the third of these areas. This qualitative study will seek to address the overarching question: *What are the enabling and limiting factors associated with implementing social prescribing, across different European countries, from the perspective of key stakeholders?*

## Methods

### Study design

Numerous qualitative studies have been published on social prescribing, from a range of countries, exploring different aspects including link workers’ contribution, patient experiences and the role of the VCS.^23^,^28^ The research outlined in this protocol is novel in adopting a cross-country qualitative analysis to explore social prescribing delivery. Taking a cross-country approach ‘offers a unique opportunity to distil diverse international perspectives into insights for health services research, policy, and practice’.^29^ The study will run from January 2026 to December 2027. Findings will be reported in line with the consolidated criteria for reporting qualitative research (COREQ).^30^

### Sampling

Five European countries will be involved—Austria, England, Germany, Poland, Portugal; some (eg, England) have a more developed national implementation of social prescribing, while for other countries (eg, Germany) its introduction into healthcare is more recent. This difference in maturity offers the opportunity for learning across countries and to reflect on taken-for-granted assumptions about how social prescribing is best delivered. Within each country, 20 interviews will be conducted. A purposive sampling approach will be used to recruit individuals aged 18 years or over with experience of the social prescribing pathway (see figure 1) as a patient, link worker, healthcare professional, VCS representative or decision-maker/policy-maker (at a local, regional or national level) (see box 1). Across the dataset (100 interviews), will we try to ensure some geographical spread (eg, rural, urban) to achieve socio-economic variation.

Box 1 **Sampling targets**

The following indicates who will be targeted in each country involved in the study:

- 8–10 national/regional/local decision-makers (eg, involved in policy development or health boards or commissioning).
- 1–2 LGBTIQ+persons.
- 1–2 refugees and first-generation immigrants (in terms of interpreters, we will mirror what is provided as part of local healthcare practice).
- 1–2 older adults living alone.
- 3–4 healthcare professionals.
- 1–2 link workers/social prescribers.
- 1–2 voluntary-community sector (VCS) representatives.

Qualitative research is not so much about quantity of data in terms of number of participants but about richness of data in relation to furthering understanding to address the research question. The size of the sample required to give that in-depth understanding is shaped by the study aim (how broad this is), how homogenous the study population is, degree of structure to topic guides, participants’ knowledge and how vocal they are likely to be and the analytical approach being adopted.^31^ These factors were considered when planning the study, with the target of 20 participants from each of the five countries decided on. This scale of data collection will be manageable for comparing and contrasting but will still allow for a range of perspectives to be explored from each country, and to have variation in terms of participant demographics.

### Recruitment

Some countries (Germany, Poland, Portugal) are involved in a trial (as part of the larger SP-EU project) of social prescribing tailored for LGBTIQ+persons, refugees and first-generation immigrants and older adults living alone. Qualitative researchers from these countries will be able to recruit individuals (professionals and patients) involved in the trial. Other recruitment routes may also be used within these countries and will be used by researchers in Austria and England (not involved in the trial). These other routes will include existing networks, services and charities; researchers will set up meetings with members of these organisations to discuss the study and recruitment targets. Recruitment invitations and participant information sheets will be passed on to potential participants by organisations or the research team either face-to-face, via email or letter. In addition, posters or advertisements will be distributed/displayed by relevant organisations, which will include a QR code where a participant information sheet can be accessed. Interested participants will directly contact the research team in their country by email or phone. If, after speaking to a researcher and having any questions answered, they wish to take part, a date for interview will be arranged.

### Data collection

Semi-structured interviews will be conducted via video or telephone, or in person; we will use the approach preferred by a participant as far as possible, to ensure that a range of people can be involved. Interviews will be conducted by researchers with experience of collecting qualitative data. A shared topic guide (with questions for each stakeholder group—see online supplemental file 1 for details) was developed collaboratively by researchers from all five countries. Included questions were based on consultation with experts in the field of social prescribing, stakeholders and literature in the field. An initial English version of the topic guide was translated into local languages by each national team and reviewed during cross-country meetings. During these meetings, researchers from the countries involved compared wording, clarified culturally specific terms and ensured conceptual equivalence through iterative discussion of semantic nuances. This resulted in a harmonised set of questions to be used across all countries—allowing for culturally appropriate adaptations when necessary. As interviews will be semi-structured, researchers will have the opportunity to follow-up on comments made by interviewees and to ask new questions if relevant. Researchers from across the five countries will meet every 2 to 3 weeks during data collection to discuss interview findings and to explore whether changes to the topic guides are required. Based on previous qualitative studies on social prescribing, it is anticipated that interviews will last between 30–60 min. Interviews will be audio-recorded, with participants’ consent.

### Analysis

**Figure 2:**
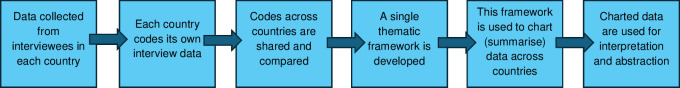
Key stages to be used in the analysis.

Figure 2 illustrates the key stages that will be undertaken to analyse the data. Interviews will be transcribed verbatim into the language in which they were conducted. Each country will code its own transcripts before translation. For the cross-country analysis, only de-identified excerpts needed for the shared thematic framework will be translated into English by the national research teams, with translations reviewed by at least one additional team member. Translation will follow an approach prioritising accuracy of meaning rather than literal wording (ie, a meaning-based translation approach). Contextual clarifications will be provided during joint analytical meetings to maintain interpretive rigour.

Framework analysis^32^ will help us to make sense of the qualitative data. It has the following interrelated stages: (1) data management (familiarisation, coding, indexing, sorting, charting) and (2) abstraction and interpretation (looking for explanations, classifications and linkages). Initially, researchers from each country will code their own data, using a computer programme with which they are familiar to support this (ie, NVivo, Atlas.ti, MAXQDA). Researchers from all five countries will then come together to explore and discuss codes, using post-it notes and miro-boards to create a thematic framework that encompasses codes from across countries. This thematic framework will be used to summarise data, in English, from all countries, into a chart using Excel. A researcher from each country will chart their own data in this shared Excel file; this will involve summarising what each participant has said (in English). In addition, a list of key quotes for each component of the thematic framework will be provided for each country—translated into English. Creating a chart that summarises data in a de-identifiable way (in Excel), using framework analysis, has been undertaken in other cross-country qualitative studies to identify commonalities and differences, and to explore possible relationships between concepts.^29 33^ Looking for differences as well as similarities will be important as the former ‘may lead to new hypotheses about explanations that inspire further studies’.^34^

### Data management

Each of the five countries involved will be responsible for storing securely their own data. Personal identifiers will be removed from transcripts before they are stored on organisational (eg, university) secure servers. Audio recordings will be deleted once checked against transcripts by a member of the research team. These data will only be accessible to appropriate members of the research team within the country holding this information. Once audio recordings have been transcribed and checked for accuracy by a member of the research team, they will be removed from the secure server.

As noted above, an Excel file will be developed that summarises de-identified data from across the five European countries. Quotations from interviews may be included but study identifiers (rather than names) will be used when sharing data across countries to avoid identification of participants. These de-identified data, in the Excel file, will be shared on KeyWays, a server being used for the wider SP-EU study.

In terms of open data, we will ask participants to consent to their anonymised data being shared for future research/analysis by the SP-EU team and beyond. Researchers wishing to use anonymised transcripts from this qualitative study, for a secondary analysis related to their own research questions, will be invited to contact the lead researchers with a request. Access to these data will be embargoed until findings have been published or a year after the SP-EU study has ended, whichever is later.

### Reflexivity

Within each country, researchers will be encouraged to take a reflexive approach to data collected and analysed, and how their background and position may shape this. Given the cross-country nature of the study, we will set aside time during team meetings involving researchers from all five countries to reflect on how different cultural backgrounds may shape data collection and interpretation.

### Potential challenges

A benefit of cross-country research is that through being exposed to different ideas and practices (eg, in terms of how healthcare is delivered, social attitudes), researchers can learn about other systems and interrogate taken-for-granted policy and practice assumptions.^33 35^ However, it can raise issues in terms of working as a multidisciplinary team, coming from different backgrounds and traditions, alongside differences in language.^29^ We have considered potential challenges related to this qualitative project, as outlined in box 2.

Box 2 **Consideration of potential challenges with this cross-country analysis**

**Language:** Heath and colleagues^29^ noted that for cross-country projects, data are often translated into English, which could make preservation of meaning difficult. For our research, each country will code its own data in its original language before selecting sections to be translated into English that are powerful or reflective of components of the thematic framework. It is recognised that this will mean we are working with decontextualised excerpts from the interviews as an overall team. However, the cross-country analysis will include researchers from each country to provide contextual details if required.

**Data quantity:** Although each country will aim to talk to the same number of people (n=20), some interviews may be longer than others. This may depend on the knowledge and involvement of interviewees in social prescribing, which could affect how verbal they are on this topic. If some countries have richer data, there is a risk of this information dominating the cross-country analysis. If this happens, we may wish to probe further into data absence by interviewing stakeholders in a country who are likely to understand the barriers and reasons why we have not been able to collect as much data there. The research team will be careful to consider concepts from across countries and not shutting down ideas early on; if an idea is only initially identified in one country, researchers from other countries will go back to their transcripts to check for its presence. It is important to highlight that Heath and colleagues^29^ did not find differences in the nature of data collected as a barrier to comparisons; they wrote that ‘size does not matter in cross-country comparative qualitative research – analytical congruency does’. Establishing analytical congruency requires time to allow for consideration of taken-for-granted assumptions across countries. We will ensure that we have at least 6 months dedicated to this cross-country comparative analysis.

**Number of countries:** Heath *et al*^29^ proposed that analysis can be particularly challenging when more than three countries are involved. This may be offset by having a narrow focus for the comparison. In addition, focusing on areas that are noticeably similar or different across countries can be helpful; adopting a framework approach and charting data will enable us to do this. Being able to reflect experiences identified in each country will call for a conceptually abstract approach to the analysis. This could elongate the time taken for the analysis but will help to ensure that this process is in-depth and considers insights from each country. It will entail thinking carefully as a team about divergence as well as overlaps between countries and incorporating these ideas into the analysis.

**Variation in perspectives of key concepts:** There may be some concepts that are difficult to translate across countries, although the fact that all are European, allowing for some similarities, should make this easier. Time will be allocated, at the start of the study, to ensure a shared understanding of key topics and social science concepts is established. All team members have a social sciences or health research background, but it will still be important to develop an agreed vocabulary to work with from the outset.

### Patient and public involvement

Co-creation is at the heart of the larger SP-EU project, playing a fundamental role throughout. For this qualitative study, we will engage with stakeholders representing the three target populations (LGBTIQ+persons, refugees and first-generation immigrants, older adults living alone) as well as those involved in social prescribing practice, policy, decision-making and research. We have engaged with stakeholders when developing this protocol and the topic guide (see online supplemental file 1). Other points at which we will engage with stakeholders include sharing: (a) an initial coding framework across the countries and (b) a draft summary of study findings – asking for reflections and comments. We will also consult with stakeholders if encountering problems as the study progresses (eg, difficulties with recruitment).

### Ethics and dissemination

Initial ethical approval was secured through the University of Oxford’s Medical Sciences Interdivisional Research Ethics Committee (IDREC 1806086). This approval covers data collection in England. Other participating countries used documents from this application to obtain approval from their respective institutional ethics committees. Approval has been obtained in Austria (through Ludwig Boltzman Gesellschaft—Ref: 033_2025), Germany (through Bergische Universität Wuppertal—Ref: SK/ats 251029) and Poland (through Wroclaw Medical University—Ref: 454/2025). In Portugal, the study protocol has been approved by the ethics committee of the National School of Public Health of the NOVA University of Lisbon; the project team is awaiting the official written confirmation document.

National teams are responsible for ensuring compliance with local institutional review procedures, General Data Protection Regulation (GDPR)-aligned data handling, secure storage on organisational servers and country-specific consent procedures. All consent materials have been translated and adapted to meet local ethical and legal requirements. All data shared for cross-country analysis will be de-identified to meet data protection standards applicable in each jurisdiction.

The study has been designed to minimise risks and burden to participants—for example, providing a clear study information sheet to each participant and having the option of how to be interviewed (video or phone conversation, or in-person). If someone experiences any distress, the interview will be stopped by the researcher and, if necessary, ended. Consent will be secured from each participant to take part, to audio-record the conversation, to share de-identified extracts of their data between researchers in the five countries involved and to use anonymised quotations from their interview in publications and presentations. In addition, they will be asked to consent to the sharing of de-identified transcripts with other researchers wishing to conduct studies on social prescribing through the secondary analysis of existing data.

We will report the study’s findings in a peer-reviewed journal. We will write a summary/policy brief for the National Academy for Social Prescribing and for the International Social Prescribing Collaborative to share with their members. For the SP-EU study’s webpage, a researcher from each country will present a short (5 min) videoed overview of this qualitative project’s findings. We will also produce an infographic that can be shared via social media. Furthermore, findings will be presented at relevant conferences.

## Supplementary material

10.1136/bmjopen-2025-112887 online supplemental file 1
