# Supplementary material for: Social prescribing within five European countries: a protocol of a cross-country qualitative analysis
Source: BMJ Open. 2026 Jan 12;16(1):e112887. doi: 10.1136/bmjopen-2025-112887 (PMC12815034; doi:10.1136/bmjopen-2025-112887)
Supplement: online supplemental file 1 [file bmjopen-16-1-s001.docx]

**Supplementary file 1: Example of types of questions to be asked to different stakeholders in interviews**

*The interview will take the form of a guided conversation. Interviewees can raise issues of importance to them. Hence, the topic guide may change as data collection progresses.*

| ***Topics*** | ***Patients*** | ***Link workers*** | ***Healthcare professionals*** | ***Voluntary-community representatives*** | ***Decision makers*** |
| --- | --- | --- | --- | --- | --- |
| **Understanding of social prescribing** | What does the term social prescribing mean to you?  [Have you been connected by a health professional (GP, nurse, social worker) to a community activity or support — like a walking group, housing support, art class — instead of medical treatment?] | What does the term social prescribing mean to you? | What does the term social prescribing mean to you? | What does the term social prescribing mean to you? | What does the term social prescribing mean to you? Where does it sit in relation to other policy/policy developments? |
| **Expectations of social prescribing** | How did you hope that meeting with [link worker] would help you? | Tell me a bit about how you came to be a link worker? What were your expectations of the role? | Tell me a bit about how you came to be involved in social prescribing? What were your expectations of your role? | What do you think social prescribing should be doing? How do you think it should support people? | What outcomes would you be looking for/expect from social prescribing? |
| **Accessibility** | How easy was it for you to access social prescribing? How were you referred to it? How did you find connecting with community activities or services? | How accessible is social prescribing for people who face additional barriers (e.g. because they do not speak the national language, experience discrimination due to their gender/sexual orientation or are older people with cognitive impairments)? | How accessible is social prescribing for people who face additional barriers (e.g. because they do not speak the national language, experience discrimination due to their gender/sexual orientation or are older people with cognitive impairments)? | How accessible is social prescribing for people who face additional barriers (e.g. because they do not speak the national language, experience discrimination due to their gender/sexual orientation or are older people with cognitive impairments)? | How accessible is social prescribing for people who face additional barriers (e.g. because they do not speak the national language, experience discrimination due to their gender/sexual orientation or are older people with cognitive impairments)? |
| **Role of link workers** | How much did you know about link workers and what they do before being referred to one? | What training have you received as a link worker? How adequate has this been in meeting your needs? | What do you think about the introduction of link workers to support people’s health and well-being? | What do you think about the introduction of link workers to support people’s health and well-being? | What do you think about the introduction of link workers to support people’s health and well-being? |
| **Interactions and collaboration** | In what ways, if any, has being involved in social prescribing changed how you relate to healthcare professionals or others in your community? | What interactions have you had with other actors (e.g. healthcare professionals, voluntary-community providers) as part of social prescribing? How would you describe these interactions – are they short-term or ongoing? If ongoing, what helps to maintain these relationships? | Can you tell me a bit about interactions you have had with link workers? How would you describe these interactions – are they short-term or ongoing? If ongoing, what helps to maintain these relationships? | Can you tell me a bit about interactions you have had with link workers? How would you describe these interactions – are they short-term or ongoing? If ongoing, what helps to maintain these relationships? | How far do you interact/engage with people involved in delivering social prescribing (e.g. link workers)? |
| **Relevance for Underserved Groups (or Tailoring and Inclusiveness)** | How do you think social prescribing can support people who are [older, refugees, LGBTQIA+ - depending on what group the interviewee is from]? | Who do you think social prescribing should be made available to? How do you think social prescribing can help people from [three groups]? | Who do you think social prescribing should be made available to? How do you think social prescribing can help people from [three groups]? | Who do you think social prescribing should be made available to? How do you think social prescribing can help people from [three groups]? | Who do you think social prescribing should be made available to? How do you think social prescribing can help people from [three groups]? |
| **Potential barriers and enablers** | What might make it hard for people to engage in or get support through social prescribing? What might make it easier for people to engage in or get support through social prescribing? | What might stop people from [three groups] engaging with and benefitting from social prescribing? What might help people from [three groups] to engage with and benefit from social prescribing? | What might stop people from [three groups] engaging with and benefitting from social prescribing? What might help people from [three groups] to engage with and benefit from social prescribing? | What might stop people from [three groups] engaging with and benefitting from social prescribing? What might help people from [three groups] to engage with and benefit from social prescribing? | What might stop or help people from [three groups] engaging with and benefitting from social prescribing? What might help people from [three groups] to engage with and benefit from social prescribing? How might social prescribing be funded in this country going forwards? |
| **Improvements** | What could be improved about social prescribing from your experience? | What can be done to improve delivery of social prescribing to people from [three groups]? | What can be done to improve delivery of social prescribing to people from [three groups]? | What can be done to improve delivery of social prescribing to people from [three groups]? | What can be done to improve delivery of social prescribing to people from [three groups]? |
| **Final reflections** | Is there anything else you would like to share about your experience of social prescribing? | Is there anything else you would like to share about your experience of social prescribing? | Is there anything else you would like to share about your experience of social prescribing? | Is there anything else you would like to share about your experience of social prescribing? | Is there anything else you would like to share about your experience of social prescribing? |
