# Supplementary material for: Social prescribing within five European countries: a protocol of a cross-country qualitative analysis
Source: BMJ Open. 2026 Jan 12;16(1):e112887. doi: 10.1136/bmjopen-2025-112887 (PMC12815034; doi:10.1136/bmjopen-2025-112887)
Supplement: Supplementary file 2 [file bmjopen-2025-112887.reviewer_comments.pdf]

PEER REVIEW HISTORY

BMJ Open publishes all reviews undertaken for accepted manuscripts. Reviewers are asked to complete a checklist review form and are provided with free text boxes to elaborate on their assessment. These free text comments are reproduced below.

ARTICLE DETAILS

Title (Provisional)

Social prescribing within five European countries: A protocol of a cross-country qualitative analysis

Authors

Tierney, Stephanie; Westlake, Debra; Rezvani, Farhad; Rojatz, Daniela; Köberlein-Neu, Juliane; Bommhardt, Trutz; Dias, Sónia; Marques, Maria João; Kurpas, Donata; Napierala, Hendrik; Herrmann, Wolfram; Husk, K

VERSION 1 - REVIEW

**Reviewer**	**1**
**Name**	**Riiser, Kirsti**
**Affiliation**	**Oslo Metropolitan University, Rehabilitation Science and Health Technology**
**Date**	**23-Nov-2025**
**COI**	**None**

Thank you for the opportunity to review this protocol, which clearly describes the design of a comprehensive, robust study with the potential to advance understanding of how social prescribing is delivered and tailored to different populations across countries. I commend the researchers for planning this important study. I only have a few questions and comments:

-Page 5, line 14: Could you please add a short overview of the translation and harmonization process for the interview guide across countries?

-Page 6, line 46-47: Please provide a short overview of the procedure for translating transcripts into English (for the framework)

-Page 6, line 56: Given the multi-country design, could you add a short overview of how ethics approvals and data protection requirements was addressed in each participating country?

VERSION 1 - AUTHOR RESPONSE

Reviewer comment	Amendment
Could you please add a short overview of the translation and harmonization process for the interview guide across countries?	*We have provided the following extra detail on page 5:*<br><br>A shared topic guide (with questions for each stakeholder group – see Supplementary file 1 for details) was developed collaboratively by researchers from all five countries. Included questions were based on consultation with experts in the field of social prescribing, stakeholders and literature in the field. An initial English version of the topic guide was translated into local languages by each national team and reviewed during cross-country meetings. During these meetings, researchers from the countries involved compared wording, clarified culturally specific terms, and ensured conceptual equivalence through iterative discussion of semantic nuances. This resulted in a harmonised set of questions to be used across all countries - allowing for culturally appropriate adaptations when necessary.
Please provide a short overview of the procedure for translating transcripts into English (for the framework)	*We have provided the following extra details on page 6:*<br><br>Interviews will be transcribed verbatim into the language in which they were conducted. Each country will code its own transcripts before translation. For the cross-country analysis, only de-identified excerpts needed for the shared thematic framework will be translated into English by the national research teams, with translations reviewed by at least one additional team member. Translation will follow an approach prioritising accuracy of meaning rather than literal wording (i.e. a meaning-based translation approach). Contextual clarifications will be provided during joint analytical meetings to maintain interpretive rigour.
Given the multi-country design, could you add a short overview of how ethics approvals and data protection requirements was addressed in each participating country?	*We have provided the following extra detail on page 7:*<br><br>Initial ethical approval was secured through the University of Oxford (IDREC 1806086). Each participating country has obtained approval from their respective institutional ethics committees, based on documents approved by the University of Oxford. National teams are responsible for ensuring compliance with local institutional review procedures, GDPR-aligned data handling, secure storage on organisational servers, and country-specific consent procedures. All consent materials have been translated and adapted to meet local ethical and legal requirements. All data shared for cross-country analysis will be de-

	identified to meet data protection standards applicable in each jurisdiction.

VERSION 2 - REVIEW

Reviewer 1

Name Riiser, Kirsti

Affiliation Oslo Metropolitan University, Rehabilitation Science and Health Technology

Date 12-Dec-2025

COI

Dear Authors,

I am satisfied with the revisions that you have made and wish you the best of luck with this comprehensive and important study. I look forward to reading the final results.
